# The pitfalls of evaluating asthma control in children and adolescents

**DOI:** 10.36416/1806-3756/e20250145

**Published:** 2025-05-21

**Authors:** Regina Maria de Carvalho-Pinto, Magali Santos Lumertz, Débora Carla Chong-Silva

**Affiliations:** 1. Divisão de Pneumologia, Instituto do Coração - InCor - Hospital das Clínicas, Faculdade de Medicina, Universidade de São Paulo, São Paulo (SP) Brasil.; 2. Escola de Medicina, Pontifícia Universidade Católica do Rio Grande do Sul, Porto Alegre (RS) Brasil.; 3. Departamento de Pediatria, Hospital Moinhos de Vento, Porto Alegre (RS) Brasil.; 4. Serviço de Alergia, Imunologia e Pneumologia Pediátrica - Complexo do Hospital de Clínicas - Universidade Federal do Paraná, Curitiba (PR) Brasil.

Asthma is one of the most common chronic diseases in childhood and adolescence. The Global Asthma Report indicates a global asthma prevalence of 9.1% among children, 11.0% among adolescents, and 6.6% among adults.^1^ ^,^ ^2^ Furthermore, according to the GINA , these numbers appear to be increasing in many countries, especially among children.^3^ Childhood asthma is also a reflection of social inequality, as the greatest burden is often associated with low-income families, even among school-aged children.^4^ In Brazil, a study based on data from the Global Burden of Disease estimated the prevalence of asthma in the pediatric population to be 12.1%.^5^ Brazilian studies have shown a progressive decline in asthma-related hospitalization rates.^6^ ^,^ ^7^ These improvements are at least partially attributed to a program for the distribution of asthma medications for free, which has been in place for approximately fifteen years, via the Brazilian *Sistema Único de Saúde* (SUS, Unified Health Care System).^6^ ^-^ ^8^ A study published in 2020 described trends of hospital admissions due to asthma between 2008 and 2015 and evaluated their relationship with trends of inhaled corticosteroids (ICS) provision by the government in Brazil. Although the decrease in hospital admissions per 100,000 population was the greatest in those with an age range of 15-39 years (from 59.9 to 32.3), a notable reduction was also observed among children and adolescents aged 5-14 years (from 148.3 to 110.9). These results reinforce that the provision of ICS free of charge by the Brazilian government was associated with a decrease in asthma-related admissions at both municipal and national levels during that period. The reduction in hospital admissions was also associated with the increase in the number of physicians and in the number of subjects who received ICS. Although asthma control using specific tools-GINA, Asthma Control Test (ACT) or Childhood ACT (cACT)-was not directly assessed, it can be inferred that the reduction in hospitalizations was due to improved care and asthma control.^9^

Assessing control is one of the main pillars of asthma treatment. It is widely recognized that effective asthma control leads to better outcomes-not only for the individual and his/her family but also for social impact. In addition to access to appropriate follow-up and treatment, one significant barrier to asthma control is the persistence of misconceptions regarding the diagnosis and management of the disease, especially in the pediatric population. In clinical practice, asthma treatment is guided by severity and adjusted on the basis of the level of control.^3^ According to a study published in 2016, caregivers of children and adolescents with asthma often show less concern about the use of systemic corticosteroids (which are typically used in exacerbations) than about aerosolized medications, particularly pressurized inhalers.^10^ This perception contributes to poor adherence to treatment and, consequently, lack of disease control.^10^ ^,^ ^11^ Moreover, it is well documented that both patients/caregivers and health care professionals tend to overestimate asthma control.^12^ ^,^ ^13^ So, determining asthma control is still a challenge on a daily basis of pediatric practice.

The Global Asthma Network Phase I, a cross-sectional study, evaluated asthma management and control in children, adolescents, and adults across 25 countries. The sample included 101,777 children and 157,784 adolescents. Among these, 6,445 (6.3%) children and 12,532 (7.9%) adolescents had physician-diagnosed asthma. For children and adolescents, asthma was assessed using written questionnaires distributed in schools. Adolescents (13-14 years old) completed self-administered questionnaires, while children (6-7 years old) questionnaires were completed by their parents. Asthma control was defined by two questions: unscheduled visits to a doctor or to the emergency department due to asthma, and hospital admissions due to asthma. Three categories were used to define asthma control: poorly controlled, partially controlled, and well-controlled asthma. Among children and adolescents, well-controlled asthma, according to the study definition, was achieved in only 44.1% of children and 55.4% of adolescents.^14^

A real-world cross-sectional study evaluating children and adolescents showed that the asthma control questionnaire (cACT/ACT) revealed that about 30% of patients perceived uncontrolled asthma. On the other hand, GINA asthma control level was considerably lower (12.6%), highlighting the difference between scores used in the assessment of asthma control.^15^ One could argue that these disparities are partly due to the self-administered nature of the cACT/ACT questionnaires, which may not always be well understood by patients.

Therefore, it is crucial to use validated tools designed to assess asthma control in the pediatric population, such as the GINA questionnaire and specific instruments such as the cACT for younger children and the ACT for adolescents ≥ 12 years of age. This was the objective of the study by Amorim et al. published in this issue of the *Jornal Brasileiro de Pneumologia*: to compare two validated instruments capable of measuring pediatric asthma control and to verify the agreement between them, as well as the agreement with clinical, laboratory, and spirometric variables.^16^

As discussed, asthma control is the central pillar in preventing exacerbations, hospitalizations, and long-term loss of lung function.^11^ The lack of comparison between the GINA and the cACT/ACT questionnaires, both widely used in outpatient settings and clinical studies, still represents a gap in the national literature.

The study stands out for demonstrating, in a representative sample of patients treated at the SUS, that the GINA questionnaire is more sensitive in identifying pediatric patients with uncontrolled asthma than are the cACT/ACT. Despite showing moderate agreement between the instruments (Kappa = 0.505), the study highlights that only GINA was able to detect significant differences in clinical (smoke exposure, disease severity, and IC dosage) and spirometric variables (FEV_1_, FVC, FEF_25-75%_) between groups classified as having controlled and uncontrolled asthma. This finding adds strength to the results and suggests that the cutoff value used by the cACT/ACT leads to an overestimation of asthma control-a fact already noted in previous international studies,^15^ but until now, little explored in the Brazilian pediatric population. Moreover, the article explores relevant contextual factors, such as the choice of inhaler device (aerosol versus dry powder), treatment adherence, and the reality of the SUS, which add not only robustness to the analysis but also practical applicability that goes beyond a simple comparison between scales.

From a practical standpoint, the findings have a direct impact on clinical management by pediatric pulmonologists, allergists, and primary care physicians. The demonstration that GINA has greater discriminatory power suggests that, in various contexts, this instrument may be more effective in guiding therapeutic decisions, optimizing drug prescriptions, and adjusting monitoring and follow-up strategies. Additionally, the association between lack of control and factors such as passive smoking exposure reinforces the need for multidisciplinary and educational approaches in the care of children with asthma.

The information provided by the study by Amorim et al.,^16^ which highlights the superiority of GINA as a screening tool for uncontrolled asthma in children, serves as a guide for public and private outpatient services and provides a basis for the construction of clearer and more efficient clinical flowcharts.
